# Character style and relational judgments in human–AI romance: trust, commitment, intimacy, and passion

**DOI:** 10.3389/fpsyg.2026.1819889

**Published:** 2026-04-30

**Authors:** Jin Niu, Weijiang She, Wenting Cheng, Xiao Dou, Xueqin Jia

**Affiliations:** 1 School of Literature and Media, Dongguan University of Technology, Dongguan, China; 2 School of Communication Engineering, Shenzhen City Polytechnic, Shenzhen, China; 3 Department of Visual Communication Design, Guangdong Polytechnic Normal University, Guangzhou, China; 4 Guangdong University of Science and Technology, Dongguan, China

**Keywords:** affective engagement, AI-generated characters, character style, gender differences, human–AI romance, relational judgment

## Abstract

**Introduction:**

As AI-generated characters become increasingly present in everyday emotional and romantic contexts, questions arise about whether they may begin to occupy relational space traditionally reserved for human partners. This study examines how character style (operationalized as holistic character conditions, each combining a distinct visual rendering, voice, and character background settings) and participant gender shape initial romantic evaluations of AI-generated and real human targets across four relational dimensions: Trust, Intimacy, Passion, and Commitment.

**Methods:**

A 4 (Character Style: 2D Anime, 3D Cartoon, Highly Humanoid, Real Human) × 2 (Participant Gender: Female, Male) mixed-design experiment was conducted with 134 Generation Z participants (72 female, 62 male; mean age = 19.91). Each participant viewed 30-s multimodal video introductions of four opposite-sex targets and rated them on a 12-item instrument adapted from automation trust scales and Sternberg’s Triangular Love Scale.

**Results:**

The results reveal two distinct patterns. On the foundational relational dimensions of Trust and Commitment, real human targets were generally rated higher than AI-generated conditions. For Trust, the real human target was rated significantly higher than all three AI conditions. For Commitment, this advantage was significant relative to the 3D Cartoon and Highly Humanoid conditions, but not relative to the 2D Anime condition. No significant Character Style × Gender interaction was found for either dimension, supporting the view that AI characters do not readily approach the relational standing of real humans on these judgments under brief initial exposure. On the affective engagement dimensions of Intimacy and Passion, significant Character Style × Gender interactions emerged: female participants reported elevated intimacy specifically toward 2D Anime targets, while male participants reported elevated passion specifically toward Highly Humanoid targets.

**Discussion:**

These findings suggest that the relational boundary between AI-generated and real human targets is dimension-specific rather than absolute — firmly held on trust, largely maintained on commitment, but more permeable on intimacy and passion. The study contributes a cross-disciplinary measurement framework that integrates HCI trust assessment with relationship psychology, and reveals a pattern of dimensional separation in which affective engagement dimensions can shift independently of foundational relational dimensions.

## 1 Introduction

The rapid development of artificial intelligence (AI) and artificial intelligence-generated content (AIGC) has expanded human–computer interaction beyond instrumental use, bringing AI companions into domains associated with emotional support, everyday companionship, and romantic imagination (Li and Zhang, 2024). Contemporary AI companionship now includes text-based chatbots, voice-based emotional support applications, virtual companions, and increasingly stylized or humanlike synthetic characters (Boine, 2023; Cheok and Zhang, 2019). Recent scholarship suggests that these systems may provide perceived social support and short-term emotional relief, while also raising concerns about emotional dependency, stigma, and overreliance on technologically mediated relationships (Ho et al., 2025; Sullins, 2012). Research on social chatbots and intelligent virtual agents further indicates that users may describe AI in explicitly relational terms, including friendship, emotional connection, and everyday psychological support (Pentina et al., 2023; Skjuve et al., 2022; Lee et al., 2021). Taken together, this literature suggests that the central question is no longer whether AI can simulate conversation, but whether it may begin to occupy relational space that has traditionally been reserved for human partners (Cheok et al., 2017; Koike et al., 2023).

A central question is whether AI-generated companions may begin to resemble human partners in foundational relational domains, particularly trust and commitment. Trust is not a unidimensional construct. In human close relationships, interpersonal trust is grounded in reciprocity, vulnerability, and expectations about another person’s agency under conditions of risk (Rempel et al., 1985; Thielmann and Hilbig, 2015). By contrast, trust in automation and AI can also take a more functional or affective form, grounded in predictability, consistency, and appropriate reliance when full understanding is impractical (Hoff and Bashir, 2015). Recent work on AI intimacy likewise suggests that users may experience trust through emotional stability, privacy, and the perception that an AI companion is reliably available and nonjudgmental (Huang and Lan, 2025). Trust is also developmental. Classic relationship research describes it as progressing from predictability to dependability and ultimately to faith, indicating that mature interpersonal trust depends on accumulated interaction history (Rempel et al., 1985). By contrast, initial and swift trust are formed under limited information and remain provisional and cognitively compressed (McKnight et al., 1998; Meyerson et al., 1996). Thin-slice research similarly shows that brief exposures can support meaningful first impressions while still reflecting condensed appraisals rather than fully developed relationship states (Ambady and Rosenthal, 1992; Carney et al., 2007). This does not make short-term trust trivial. Early trust judgments still matter because they shape whether a target appears credible, safe, and worth further relational investment (McKnight et al., 1998; Wilson and Cunliffe, 2022). In this context, the Uncanny Valley hypothesis remains relevant. The classic account suggests that characters falling close to—but not fully achieving—human appearance may evoke discomfort, much like lifelike dolls or humanoid robots that look almost real but carry subtle perceptual inconsistencies (Mori, 1970; Ambady and Rosenthal, 1992). This discomfort has been linked to reduced trust and avoidance responses toward near-human entities (Arsenyan and Mirowska, 2021), raising questions about whether highly humanoid AI characters might similarly affect early relational judgments. However, recent evidence suggests that this pattern is more nuanced than the classic uncanny-valley account implies. More realistic AI avatars are not always perceived as less trustworthy; in some contexts, they are judged as more trustworthy than stylized ones (Baake et al., 2025), and eeriness may peak at medium rather than high levels of realism (Tao et al., 2025). That said, these findings come mainly from task-oriented or general social evaluation settings, and whether they apply when AI characters are evaluated as potential romantic partners alongside a real human remains an open question. Thus, contemporary AI avatars and AIGC-generated characters require renewed investigation rather than straightforward interpretation through earlier uncanny-valley assumptions. In human romantic relationships, commitment is especially important because it marks a shift from immediate attraction or emotional attachment to a more durable orientation toward continuity, responsibility, and long-term partnership (Sternberg, 1986; Sternberg, 1997). Related work has similarly conceptualized commitment in terms of dedication and constraint, emphasizing that stronger commitment judgments imply not only liking or attachment, but also willingness to accept obligation, durability, and future-oriented responsibility (Stanley and Markman, 1992). At the same time, a growing body of research suggests that AI companions are increasingly experienced not merely as tools, but as relationally meaningful partners that may occupy forms of emotional and romantic significance once more closely associated with human others (Skjuve et al., 2022; Li and Zhang, 2024). Recent studies further indicate that users may describe interactions with AI in explicitly romantic terms and, in some cases, imagine these systems as potential partners rather than purely instrumental technologies (Song et al., 2022; Ho et al., 2025). Thus, it becomes especially important to examine whether AI-generated characters remain primarily within the domain of emotional companionship, or whether they are beginning to cross relational boundaries and enter a space more typically reserved for real human partners. From this perspective, commitment offers an initial way to test whether AI-generated characters are evaluated only as sources of affective attachment or also as candidates for more consequential forms of partnership.

A second concern is whether AI-generated companions may become increasingly attractive or even habit-forming, thereby deepening emotional reliance on technology. Existing research suggests that emotional interaction with chatbots does not simply provide momentary support, but can also foster affective attachment, repeated engagement, and even dependence-like patterns in human–AI relationships (Chen et al., 2025; Ramadan, 2021; Xie et al., 2023; Guerreiro and Loureiro, 2023). Recent work on human–AI intimacy further indicates that such relationships are often layered and context-dependent: users may experience trust, attachment, or habitual reliance even when deeper emotional intimacy remains limited (Huang and Lan, 2025). This concern may be particularly salient for younger users. Research with adolescents and college students suggests that younger people are more likely to turn to social AI for companionship or emotional coping, and that such use is often associated with loneliness, lower perceived social support, or depression, indicating a heightened tendency to rely on these systems when offline support feels insufficient (Herbener and Damholdt, 2025; Vanhoffelen et al., 2025; Lai et al., 2025). Within this broader concern, intimacy and passion are especially relevant because prior research on human–AI interaction increasingly treats them as two important dimensions of users’ affective responses to AI companions (Song et al., 2022; Pal et al., 2023a, b). Existing studies also suggest that these dimensions are not merely transient reactions. In broader relationship research, intimacy may deepen over time and contribute to the later development of commitment (Acker and Davis, 1992; Bügel et al., 2011), while in AI contexts intimacy and passion have likewise been linked to stronger commitment and continued use (Song et al., 2022; Pal et al., 2023a, b). Related work on virtual intimacy further shows that richer communicative channels and more expressive interaction can strengthen perceived closeness in human–agent interaction (Potdevin et al., 2021). Against this background, character style becomes especially relevant. Research on virtual human rendering indicates that presentation style shapes how users interpret attractiveness, expressiveness, and social meaning (Stuart et al., 2022), while work on virtual romance and synthetic intimacy suggests that human likeness and anthropomorphic legibility influence whether a character is perceived as a potential relational partner rather than simply as a functional tool (Koike et al., 2023; Xie and Pentina, 2022). Taken together, this literature suggests that intimacy and passion are especially likely to vary across character styles, making them useful dimensions for examining whether AI-generated romantic characters function primarily as affective stimuli or begin to support stronger forms of emotional reliance.

Overall, the present study focuses on two narrower but related concerns in the literature on AI companionship. The first concerns whether AI-generated romantic characters can approach real humans on foundational relational judgments, particularly trust and commitment. The second concerns whether different character styles may differentially intensify affective engagement, especially intimacy and passion, in ways that may be relevant to user engagement and emotional reliance. Although prior studies have shown that users can form meaningful emotional bonds with AI companions, much of the existing work has focused on text-based interaction, virtual agents without a real human comparison condition, or general anthropomorphic acceptance rather than differentiated romantic evaluations across multiple character styles (Lee et al., 2021; Song et al., 2022; Ho et al., 2025). This leaves an important gap. It remains unclear whether graded styles of synthetic partners function mainly as affective stimuli, as potential relational substitutes, or as something in between. A design that compares 2D Anime, 3D Cartoon, Highly Humanoid, and Real Human targets therefore makes it possible to examine how character style relates differently to these four dimensions in initial romantic evaluations.

As noted above, each character style condition represents a holistic stimulus configuration (combining visual rendering, voice, and character background settings) rather than an isolated manipulation of a single visual variable. The research questions and hypotheses below should therefore be interpreted as comparisons across these integrated conditions. More specifically, this study addresses the following research questions:

RQ1: To what extent do AI-generated character style conditions differ from a real human target in trust and commitment?

RQ2: How do different character style conditions shape intimacy and passion in initial romantic evaluations?

RQ3: Do these effects vary by participant gender under the present stimulus configuration?

Based on these questions, four hypotheses were proposed:

*H1*: Real Human targets will receive higher trust ratings than AI-generated character style conditions.

*H2*: Real Human targets will receive higher commitment ratings than AI-generated character style conditions.

*H3*: Character style conditions will significantly influence intimacy and passion.

*H4*: The effects of character style conditions on intimacy and passion will vary by participant gender under the present stimulus set.

## 2 Materials and methods

### 2.1 Participants

A total of 134 valid participants were recruited for this study through an online survey platform. The sample consisted of 72 females and 62 males, ensuring a relatively balanced gender distribution. The participants’ ages ranged from 17 to 24 years old, with an average age of 19.91 years (*SD* = 1.39). This demographic represents the typical Generation Z cohort, who are the primary active users and target audience for AIGC applications and virtual companions. All participants volunteered for the study, provided explicit informed consent prior to the experiment, and were informed of their right to withdraw at any time. The studies involving human participants were reviewed and approved by Ethics Committee of Dongguan University of Technology.

### 2.2 Experimental design

**Figure 1:**
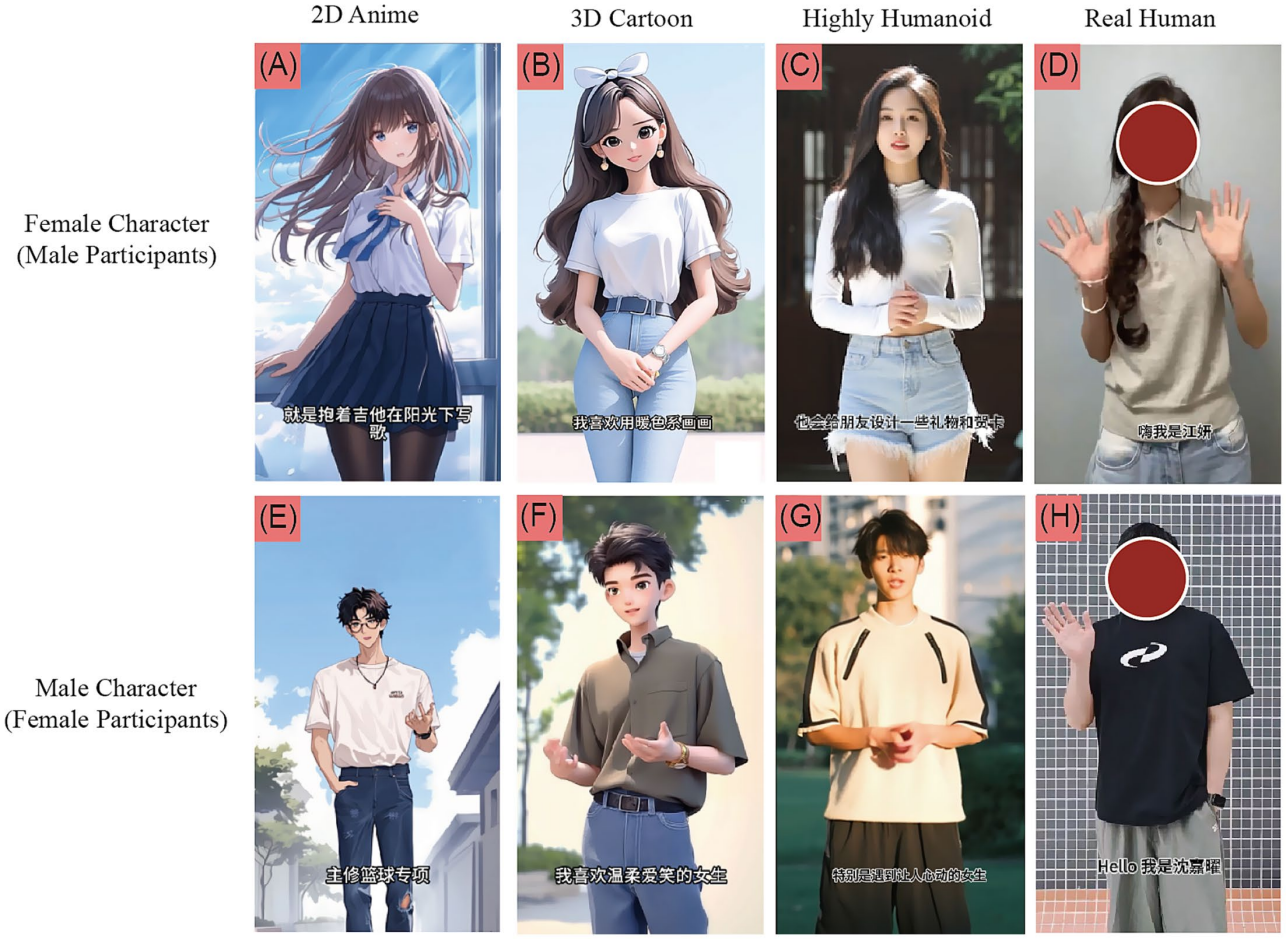
Stimulus examples for the four character style conditions. Top row **(A–D)**: female targets evaluated by male participants; bottom row (E–H): male targets evaluated by female participants. Columns from left to right: 2D Anime, 3D Cartoon, Highly Humanoid, and Real Human. Faces in the Real Human condition **(D,H)** have been pixelated to protect participant privacy.

This study employed a 4 × 2 mixed experimental design to investigate how character style and participant gender shape initial romantic evaluations, as illustrated in Figure 1. The within-subjects factor was Character Style, consisting of four levels arranged along a gradient of visual realism: 2D Anime, 3D Cartoon, Highly Humanoid, and Real Human (Karhulahti and Välisalo, 2020; Leo-Liu, 2023; Boine, 2023; Stuart et al., 2022). Each participant evaluated all four styles. The between-subjects factor was Participant Gender (Female vs. Male); participants viewed only opposite-sex targets to align the task with heterosexual romantic evaluation. The dependent variables were self-reported scores on four dimensions: Trust, Intimacy, Passion, and Commitment (Table 1). Following the two-concern framing outlined in the Introduction, Trust and Commitment serve as indicators of whether AI-generated characters approach foundational human relational judgments, whereas Intimacy and Passion serve as indicators of affective engagement relevant to emotional reliance and technology dependence.

**Table 1:** Measurement instrument.

Demographics	Item	Question	Adapted from
	Gender	What is your gender?	—
	Age	Please enter your age	—
Construct			
Trust	T1	I believe what he/she says.	Jian et al. (2000), Hoff and Bashir (2015), Schaefer (2016), Thielmann and Hilbig (2015)
	T2	I think he/she is a trustworthy character.	
	T3	I believe that I can build a relationship of mutual trust with this character in the future.	
Intimacy	ITM1	I feel comfortable and close when communicating with him/her.	Sternberg (1986, 1997), Song et al. (2022), Li and Zhang (2024), Skjuve et al. (2022)
	ITM2	I believe that I can develop an emotionally close and mutually understanding relationship with him/her in the future.	
	ITM3	I want to become good friends or soulmates with him/her.	
Passion	PS1	I find his/her figure and appearance very attractive.	Sternberg (1986, 1997), Pal et al., 2023a,b, Ho et al. (2025), Li et al. (2026)
	PS2	I believe that I can build a highly romantic and passionate relationship with him/her in the future.	
	PS3	If circumstances allow, I would want to have a physical relationship with him/her.	
Commitment	CM1	I think he/she is an ideal marriage partner.	Sternberg (1986, 1997), Song et al. (2022), Ho et al. (2025), Stanley and Markman (1992)
	CM2	I believe it is possible to enter into a committed, responsible, long-term romantic relationship with him/her.	
	CM3	If circumstances allow, I would be willing to legally marry him/her and assume the corresponding responsibilities.	

All items were measured on a 7-point Likert scale (1 = “Strongly Disagree” to 7 = “Strongly Agree”).

Each stimulus consisted of a 30-s multimodal video introduction, approximating the kind of thin-slice encounter from which meaningful first impressions can be reliably formed (Ambady and Rosenthal, 1992; Carney et al., 2007). The trust and commitment ratings obtained here should therefore be understood as initial dispositional appraisals—reflecting whether participants perceived a target as credible and worth further relational investment—rather than as indicators of established interpersonal trust or mature commitment (McKnight et al., 1998; Rempel et al., 1985). This distinction is revisited in the Discussion.

A central design consideration was that the study compares AI-generated characters against a real human baseline. This comparison inherently prevents full cross-condition equivalence of non-visual variables, because real humans carry irreducible individual features—such as natural voice timbre, spontaneous micro-expressions, and idiosyncratic mannerisms—that cannot be replicated by or imposed onto synthetic characters without distorting what each style naturally represents. The same principle applies across all character styles: 2D anime characters are conventionally associated with softer vocal qualities and exaggerated expressiveness, 3D cartoon characters tend to convey greater maturity than 2D styles, and highly humanoid avatars approximate but do not fully reproduce human expressive range (Stuart et al., 2022). Research on cross-modal perception further demonstrates that mismatching the realism level of a character’s appearance and voice produces uncanny-valley effects and reduces perceived warmth and trust (Mitchell et al., 2011; Nass and Gong, 1999), indicating that forcing uniform auditory and behavioral features across styles that differ in visual realism would introduce its own confound. More broadly, studies that simultaneously manipulate rendering style, voice quality, and animation quality in virtual agents consistently find that these channels interact, such that the perceptual impact of any single modality depends on the configuration of the others (Parmar et al., 2022). Accordingly, the design prioritized within-style multimodal coherence—ensuring that each character condition was internally consistent in voice, profile, and visual presentation—over strict cross-condition isolation of a single variable. The observed effects should therefore be interpreted as responses to holistic character conditions rather than to visual realism alone, and any gender-related differences may partly reflect the specific stimulus configurations used in the present study. The rationale for the specific profile and voice assignments is detailed in the Stimuli and Apparatus section, and the interpretive constraints they impose are addressed in the Limitations.

### 2.3 Stimuli and apparatus

The experimental stimuli consisted of eight short video clips, each approximately 30 s in duration, representing the 4 (Character Style) × 2 (Target Gender) conditions. Table 2 summarizes the character assignments, profile cues, and voice configurations for each condition.

**Table 2:** Character mapping and stimulus configuration.

Character style	Female targets (evaluated by male participants)	Male targets (evaluated by female participants)
2D Anime	Lin Zhiyao — Music Production	Zhou Zechuan — Basketball Specialization
	*Luvvoice TTS: Xiaoyi*	*Luvvoice TTS: Yunxia*
3D Cartoon	Qiao Qingqing — Oil Painting	Li Yanzhi — Track and Field Specialization
	*Luvvoice TTS: Xiaoxiao*	*Luvvoice TTS: Yunjian*
Highly humanoid	Su Shengsheng — Visual Communication Design	Song Xiao — Volleyball Specialization
	*Luvvoice TTS: Xiaobei*	*Luvvoice TTS: Yunxi*
Real human	Jiang Yan — Photography and Video Art	Shen Jiayao — Fitness and Rehabilitation
	*Natural voice recording by human actors*	*Natural voice recording by human actors*

Female characters were assigned art-related majors; male characters were assigned sports-related majors. Each participant evaluated only opposite-sex targets. TTS voice models were selected to maintain within-style multimodal coherence (see text).

The background profiles and dialogue scripts for all characters were uniformly generated using the DeepSeek-V3 large language model to ensure linguistic consistency in word count and emotional valence across conditions (see Appendix 1). Female characters were assigned art-related majors and male characters sports-related majors, providing each target with a recognizable social identity. As noted in the Experimental Design section, this configuration means that observed effects should be interpreted as responses to holistic character conditions rather than to any single isolated variable.

Auditory stimuli for the three AIGC conditions were synthesized using Luvvoice Text-to-Speech. Critically, a different TTS voice model was selected for each character style to preserve multimodal coherence along the realism gradient: the 2D Anime condition used lighter, more youthful voice models (*xiaoyi*/*yunxia*) consistent with anime character conventions; the 3D Cartoon condition used moderately warm and clear models (*xiaoxiao*/*yunjian*); and the Highly Humanoid condition used the most naturalistic available models (*xiaobei*/*yunxi*), approaching human vocal quality. This graduated voice selection follows the design principle that matching the realism level of appearance and voice within each condition avoids the cross-modal eeriness and reduced trust that arise from face–voice mismatch (Mitchell et al., 2011; Nass and Gong, 1999). Audio for the Real Human condition was recorded naturally by human actors reading the identical scripts, preserving the ecological authenticity of the human baseline.

The visual components for the three AIGC conditions were initially developed as high-quality static images and subsequently animated into 30-s videos using Keling AI (2.0). To control extraneous visual variables, a rigorous standardization protocol was applied across all stimuli. Non-verbal communication cues were standardized by requiring all characters—including the real human actors—to perform a controlled sequence of movements consisting of an initial greeting gesture followed by two non-meaningful auxiliary gestures, informed by research on the relationship between character movement and perceived personality traits (Niu et al., 2022). Environmental parameters were unified, with all stimuli simulating consistent daytime outdoor natural lighting. Characters wore casual, solid-colored clothing against neutral walls or clean campus settings to minimize visual distraction. For the Real Human condition, two college students were recruited as actors. Their physical attractiveness was carefully moderated to reflect a relatable, everyday level, avoiding extremes such as celebrity-level features or noticeable physical anomalies. Finally, while the original stimuli featured clear facial expressions, images presented in Figure 1 of this manuscript have been pixelated to comply with volunteer privacy protocols, a procedure explicitly approved by the participants.

### 2.4 Manipulation check

To verify that the four character conditions were perceived as occupying distinct positions along the intended realism gradient, an independent pilot study (*N* = 30; 15 females, 15 males) was conducted prior to the main experiment. Participants evaluated the perceived realism of each character style (“How real do you perceive this character to be?”) on a 7-point scale (1 = *completely virtual*, 7 = *completely real*). Because the pilot stimuli were presented in the same multimodal format used in the main experiment—including voice, profile, and visual appearance—the ratings reflect the perceived realism of the overall character presentation rather than visual appearance in isolation.

A 4 (Character Style) × 2 (Participant Gender) mixed-design repeated measures ANOVA revealed a highly significant main effect of character style on perceived realism, *F* (3, 84) = 130.72, *p* < 0.001, $\eta_{p}^{2}$ = 0.78. The interaction between character style and participant gender was not significant, *F* (3, 84) = 2.28, *p* = 0.085, indicating that the perceived realism gradient was consistent across male and female participants. *Post hoc* pairwise comparisons with Bonferroni correction confirmed a step-wise realism hierarchy. The perceived realism scores for 2D Anime (*M* = 3.07) and 3D Cartoon (*M* = 3.50) were relatively close, reflecting their shared non-photorealistic nature, but a statistically significant difference was nonetheless detected (*p* = 0.019). The remaining comparisons were clearly distinguished: the Real Human condition (*M* = 6.20) was perceived as significantly more realistic than the Highly Humanoid condition (*M* = 4.70, *p* < 0.001), which in turn was rated significantly more realistic than 3D Cartoon (*p* < 0.001). These results confirm that the four character conditions formed the intended realism gradient, providing a validated basis for examining how character style relates to initial romantic judgments across the four dependent variables.

### 2.5 Measures

The measurement instrument was a study-specific adaptation designed to assess initial romantic evaluations across four dimensions: Trust, Intimacy, Passion, and Commitment (Table 1). Each dimension was measured with three items on a 7-point Likert scale (1 = *Strongly Disagree*, 7 = *Strongly Agree*). The instrument draws on two bodies of literature and was tailored to the context of evaluating both AI-generated and real human targets after brief multimodal exposure.

The Trust items were adapted from established automation and interpersonal trust scales (Jian et al., 2000; Hoff and Bashir, 2015; Schaefer, 2016; Thielmann and Hilbig, 2015). Because the present study examines responses to characters that function as potential social partners rather than as purely instrumental systems, the items were reformulated to assess perceived credibility (T1), character trustworthiness (T2), and the potential for mutual trust (T3), bridging the automation trust literature and the interpersonal trust tradition discussed in the Introduction.

The Intimacy, Passion, and Commitment items were adapted from Sternberg’s Triangular Love Scale (Sternberg, 1986, 1997), with modifications informed by recent research on human–AI romantic interaction. The Intimacy items capture perceived emotional closeness (ITM1), the potential for mutual understanding (ITM2), and the desire for deeper relational connection (ITM3), reflecting evidence that users may describe AI companions in terms of friendship, emotional support, and soulmate-like bonding (Li and Zhang, 2024; Skjuve et al., 2022; Song et al., 2022). The Passion items assess visual and physical attraction (PS1), romantic projection (PS2), and desire for physical connection (PS3), dimensions that have been increasingly documented in cyber-psychology research on human–AI relationships (Ho et al., 2025; Li et al., 2026; Pal et al., 2023a, b).

The Commitment items require particular explanation because they employ strong real-world relational concepts—including “ideal marriage partner” (CM1) and “legally marry” (CM3)—that may appear disproportionate to a 30-s encounter. This was a deliberate design choice. As outlined in the Introduction, the first concern of the present study is whether AI-generated characters approach real humans on foundational relational judgments, particularly commitment. To address this question, the items were designed as a boundary test: by applying the strongest available relational benchmark, they make it possible to identify whether participants are willing to extend consequential relational evaluations—such as long-term partnership and legal obligation—to AI-generated targets, or whether such evaluations remain reserved for real humans (Ho et al., 2025; Stanley and Markman, 1992). A low or floor-level response to these items under AIGC conditions is therefore not a measurement failure but a theoretically informative outcome, revealing the boundary at which relational extension toward artificial characters ceases. This rationale was introduced in the theoretical framing and is revisited in the Discussion.

The resulting four-construct instrument is best understood as a study-specific adaptation rather than a direct deployment of any single existing scale. The construct validity and internal consistency of the instrument are reported in the Results section.

### 2.6 Procedure

The experimental procedure was administered via the “Wenjuanxing” online platform, which facilitated the seamless integration of high-definition video stimuli into the survey environment. Upon accessing the experimental link, participants were presented with a formal interface detailing the study’s objectives, data confidentiality protocols, and their right to voluntary withdrawal. After providing informed consent, participants completed a brief demographic section covering age and gender. To mitigate potential order effects—such as carry-over effects or cognitive fatigue—the presentation sequence of the four character conditions followed a strictly randomized order generated by a Latin Square design. Within the experimental core, participants were required to watch each 30-s stimulus video in its entirety before the evaluation items were activated, ensuring that ratings reflected full exposure to the multimodal character presentation. Following each viewing, the 12-item measurement instrument was administered to assess the four dimensions of initial romantic evaluation: Trust, Intimacy, Passion, and Commitment. This sequential “observation–evaluation” cycle was repeated until all four character conditions had been assessed. Upon successful completion, participants were debriefed and provided with a compensatory payment of 20 RMB.

### 2.7 Data analysis

All statistical analyses were conducted using JASP (Version 0.19.2; JASP Team, 2024). The analytic strategy proceeded in three stages, each addressing a distinct purpose.

#### 2.7.1 Stage 1: measurement quality

Before testing substantive hypotheses, the psychometric properties of the 12-item instrument were evaluated. Internal consistency was assessed with both Cronbach’s *α* and McDonald’s *ω* to provide complementary estimates of reliability (Goss-Sampson, 2019). Confirmatory factor analysis (CFA) was then conducted to verify that the four proposed constructs—Trust, Intimacy, Passion, and Commitment—were empirically distinguishable. Inter-construct correlations were examined to confirm that, although the four dimensions are conceptually related—three adapted from Sternberg's (1986) love theory and recent human–AI interaction research, and Trust from the automation and interpersonal trust literature—their correlations remain below conventional multicollinearity thresholds, supporting their treatment as separate outcomes.

#### 2.7.2 Stage 2: hypothesis testing

The primary analyses consisted of four separate 4 (Character Style) × 2 (Participant Gender) mixed-design ANOVAs, one for each dependent variable. Character Style was treated as the within-subjects factor and Participant Gender as the between-subjects factor. Each ANOVA addressed specific hypotheses: Trust and Commitment were analyzed to test H1 and H2 (whether the Real Human condition is rated higher than AIGC conditions), while Intimacy and Passion were analyzed to test H3 and H4 (whether character style and gender interact to shape affective engagement).

The decision to analyze the four dimensions separately rather than in a single multivariate model was made for three reasons. First, the two-concern framing of the study treats Trust and Commitment as foundational relational indicators and Intimacy and Passion as affective engagement indicators; collapsing them into a single omnibus test would obscure the dimension-specific patterns that are central to answering the research questions. Second, each hypothesis makes directional predictions about a specific dependent variable, and separate ANOVAs allow these predictions to be tested transparently. Third, although the CFA confirms that the four constructs are correlated, they are theoretically distinct—three grounded in Sternberg's (1986) love model and recent human–AI research, and one in the trust literature—and the inter-construct correlations reported below remain well below multicollinearity thresholds, supporting independent analysis.

Sphericity assumptions were verified using Mauchly’s test, with Greenhouse–Geisser corrections applied where violated. Significant main effects of Character Style were followed up with *post hoc* pairwise comparisons using Bonferroni adjustment. Where the Character Style × Gender interaction was significant, simple effects analyses were conducted to decompose the interaction and identify the source of gender-specific patterns.

#### 2.7.3 Stage 3: descriptive overview

Means and standard deviations across all experimental conditions were computed and are reported alongside the inferential results to provide a full picture of the data.

## 3 Results

### 3.1 Measurement quality

Internal consistency and construct validity were evaluated before testing the substantive hypotheses. As shown in Table 3, all four constructs demonstrated excellent reliability, with Cronbach’s *α* ranging from 0.932 to 0.958 and McDonald’s *ω* ranging from 0.933 to 0.958, all well above the 0.70 threshold.

**Table 3:** Reliability and construct validity of the measurement model.

Constructs	Items	Standardized factor loadings	Cronbach’s *α*	McDonald’s *ω*
Trust	T1	0.885	0.932	0.933
	T2	0.945		
	T3	0.929		
Intimacy	ITM1	0.896	0.941	0.942
	ITM2	0.961		
	ITM3	0.929		
Passion	PS1	0.898	0.946	0.946
	PS2	0.971		
	PS3	0.939		
Commitment	CM1	0.942	0.958	0.958
	CM2	0.953		
	CM3	0.937		

*N* = 134. All standardized factor loadings are highly significant (*p* < 0.001). T: Trust; ITM: Intimacy; PS: Passion; CM: Commitment.

A confirmatory factor analysis (CFA) was conducted on the full sample (*N* = 134) to verify the four-factor measurement structure. The model showed an excellent fit to the data: *χ*^2^ (48) = 157.97, *p* < 0.001, CFI = 0.997, TLI = 0.995, SRMR = 0.022, RMSEA = 0.065. All standardized factor loadings ranged from 0.885 to 0.971 and were highly significant (*p* < 0.001), indicating strong convergent validity (Table 3).

Descriptive statistics and Pearson correlation coefficients for the four constructs are presented in Table 4. The mean scores ranged from 3.57 (*SD* = 1.87) for Commitment to 3.92 (*SD* = 1.58) for Trust. All inter-construct correlations were positive and significant (*p* < 0.001), with coefficients ranging from *r* = 0.498 to *r* = 0.652. These correlations indicate that the four dimensions are related, as would be expected for constructs drawn from overlapping relational and evaluative domains. At the same time, all coefficients remained well below the 0.85 threshold commonly used to indicate multicollinearity, confirming that the constructs are sufficiently distinct to be treated as separate dependent variables in the subsequent analyses.

**Table 4:** Descriptive statistics and inter-construct correlations.

Constructs	Mean	SD	1. Trust	2. Intimacy	3. Passion	4. Commitment
1. Trust	3.92	1.58	—			
2. Intimacy	3.88	1.73	0.498***	—		
3. Passion	3.71	1.89	0.503***	0.560***	—	
4. Commitment	3.57	1.87	0.616***	0.568***	0.652***	—

*N* = 134. ***p < 0.001.

### 3.2 Foundational relational judgments: trust and commitment

The first set of analyses addressed H1 and H2 by examining whether the Real Human condition was rated higher than the three AIGC conditions on Trust and Commitment—the two dimensions treated as foundational relational indicators. Descriptive statistics for all conditions are presented in Table 5 and the full ANOVA results in Table 6.

**Table 5:** Descriptive statistics of the four dimensions across character styles and gender.

Style	Gender	Trust *(M ± SD)*	Intimacy *(M ± SD)*	Passion *(M ± SD)*	Commitment *(M ± SD)*
2D	Female	3.61 ± 1.56	4.32 ± 1.97	3.37 ± 1.69	3.07 ± 1.95
	Male	4.00 ± 1.52	4.13 ± 1.85	4.00 ± 2.07	4.12 ± 1.86
	Total	3.79 ± 1.55	4.23 ± 1.91	3.66 ± 1.90	3.56 ± 1.97
3D Cartoon	Female	3.38 ± 1.63	3.38 ± 1.71	3.09 ± 1.90	2.89 ± 1.85
	Male	4.04 ± 1.33	3.94 ± 1.75	4.04 ± 1.64	3.80 ± 1.68
	Total	3.69 ± 1.53	3.64 ± 1.75	3.53 ± 1.84	3.31 ± 1.83
Highly humanoid	Female	3.63 ± 1.54	3.86 ± 1.79	3.23 ± 1.67	3.04 ± 1.78
	Male	4.12 ± 1.50	3.99 ± 1.41	5.35 ± 1.74	3.97 ± 1.58
	Total	3.86 ± 1.54	3.92 ± 1.62	4.21 ± 2.00	3.47 ± 1.74
Real human	Female	4.05 ± 1.78	3.41 ± 1.60	3.00 ± 1.78	3.44 ± 2.18
	Male	4.72 ± 1.38	4.11 ± 1.51	3.91 ± 1.59	4.55 ± 1.28
	Total	4.36 ± 1.64	3.74 ± 1.59	3.42 ± 1.75	3.95 ± 1.90

**Table 6:** Results of the mixed-design repeated measures ANOVA.

Construct	Character style		Participant gender		Character style × participant gender	
	*F* (3, 396)	$\eta_{p}^{2}$	*F* (1, 132)	$\eta_{p}^{2}$	*F* (3, 396)	$\eta_{p}^{2}$
Trust	8.400***	0.06	7.747**	0.055	0.43	0.003
Intimacy	5.110**	0.037	1.743	0.013	3.492*	0.026
Passion	10.753***	0.075	24.340***	0.156	8.484***	0.06
Commitment	7.369***	0.053	15.070***	0.102	0.237	0.002

Mauchly’s test indicated a violation of sphericity; Greenhouse–Geisser corrected p-values are reported (Intimacy ε = 0.941; Passion ε = 0.942; Commitment ε = 0.929). *p < 0.05. **p < 0.01. ***p < 0.001.

For trust dimension, Mauchly’s test indicated that the sphericity assumption was not violated (*p* = 0.072). A significant main effect of Character Style was found, *F* (3, 396) = 8.400, *p* < 0.001, $\eta_{p}^{2}$ = 0.060. *Post hoc* comparisons with Bonferroni correction showed that Real Human targets (*M* = 4.36, *SD* = 1.64) were rated significantly more trustworthy than 2D Anime (*M* = 3.79, *SD* = 1.55, *p* = 0.003), 3D Cartoon (*M* = 3.69, *SD* = 1.53, *p* < 0.001), and Highly Humanoid targets (*M* = 3.86, *SD* = 1.54, *p* = 0.002). No significant differences were found among the three AIGC conditions (all *p*s > 0.05). A significant main effect of Gender was also observed, *F* (1, 132) = 7.747, *p* = 0.006, $\eta_{p}^{2}$ = 0.055, with male participants reporting higher trust scores than female participants across all conditions. The Character Style × Gender interaction was not significant, *F* (3, 396) = 0.430, *p* = 0.732, $\eta_{p}^{2}$ = 0.003, indicating that the trust advantage for Real Human targets was consistent across both genders. These results support H1: Real Human targets will receive higher trust ratings than AI-generated character styles.

For commitment dimension, Mauchly’s test indicated a violation of sphericity (*p* = 0.016); Greenhouse–Geisser corrected *p*-values are reported (*ε* = 0.929). A significant main effect of Character Style was found, *F* (3, 396) = 7.369, *p* < 0.001, η^2^*p* = 0.053. Post hoc comparisons showed that Real Human targets (*M* = 3.95, *SD* = 1.90) received significantly higher commitment ratings than 3D Cartoon (*M* = 3.31, *SD* = 1.83, *p* < 0.001) and Highly Humanoid targets (*M* = 3.47, *SD* = 1.74, *p* = 0.002). The difference between Real Human and 2D Anime (*M* = 3.56, *SD* = 1.97) approached but did not reach significance (*p* = 0.087). No significant differences were found among the three AIGC conditions (all *p*s > 0.524). A significant main effect of Gender was observed, *F* (1, 132) = 15.07, *p* < 0.001, $\eta_{p}^{2}$ = 0.102, with male participants again reporting higher commitment scores. The Character Style × Gender interaction was not significant, *F* (3, 396) = 0.237, *p* = 0.857, $\eta_{p}^{2}$ = 0.002, confirming that the commitment pattern did not differ between genders. These results largely support H2, though the non-significant difference between Real Human and 2D Anime suggests that the boundary may be less clear-cut for this particular comparison.

Overall, for both Trust and Commitment, the Real Human condition was rated significantly higher than at least two of the three AIGC conditions, and neither dimension showed a significant Style × Gender interaction. This pattern is consistent with the first concern outlined in the Introduction: AI-generated characters, regardless of their visual style, did not approach the Real Human baseline on foundational relational judgments in the present brief-exposure paradigm. Gender influenced the overall level of ratings but not the relative ordering of character conditions.

### 3.3 Affective engagement: intimacy and passion

The second set of analyses addressed H3 and H4 by examining whether character style influenced Intimacy and Passion and whether these effects varied by participant gender. Both Intimacy and Passion violated the sphericity assumption (Intimacy: Mauchly’s *p* = 0.035, ε = 0.941; Passion: Mauchly’s *p* = 0.026, ε = 0.942); Greenhouse–Geisser corrected *p*-values are reported for all within-subjects effects.

For Intimacy, a significant main effect of Character Style was found, *F* (3, 396) = 5.110, *p* = 0.002, $\eta_{p}^{2}$ = 0.037. The main effect of Gender was not significant, *F* (1, 132) = 1.743, *p* = 0.189, $\eta_{p}^{2}$ = 0.013. Critically, the Character Style × Gender interaction was significant, *F* (3, 396) = 3.492, *p* = 0.018, $\eta_{p}^{2}\;$ = 0.026, indicating that the effect of character style on intimacy differed between female and male participants.

**Figure 2:**
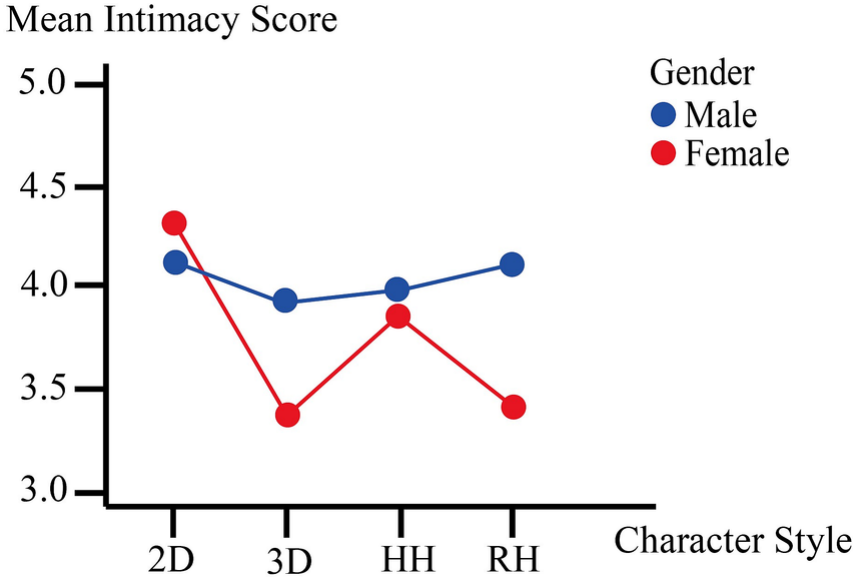
Interaction effect of character style × participant gender on intimacy. 2D = 2D anime; 3D = 3D cartoon; HH = highly humanoid; RH = real human.

Simple effects analyses revealed that character style significantly influenced intimacy ratings among female participants, *F* (3, 396) = 11.832, *p* < 0.001. Conditional pairwise comparisons showed that female participants rated 2D Anime targets (*M* = 4.32, *SD* = 1.97) significantly higher in intimacy than both 3D Cartoon (*M* = 3.38, *SD* = 1.71, *p* < 0.001) and Real Human targets (*M* = 3.41, *SD* = 1.60, *p* = 0.001). The difference between 2D Anime and Highly Humanoid (*M* = 3.86, *SD* = 1.79) did not reach significance (*p* = 0.125). No other pairwise differences were significant among female participants. By contrast, no significant simple effect of character style was found among male participants, *F* (3, 396) = 0.260, *p* = 0.854, indicating that male participants reported similar levels of intimacy across all four character conditions (Figure 2).

For passion, a significant main effect of Character Style was found, *F* (3, 396) = 10.753, *p* < 0.001, $\eta_{p}^{2}\;$ = 0.075. A significant main effect of Gender was also observed, *F* (1, 132) = 24.34, *p* < 0.001, $\eta_{p}^{2}\;$ = 0.156, with male participants reporting higher passion scores than female participants across conditions. Critically, the Character Style × Gender interaction was highly significant, *F* (3, 396) = 8.484, *p* < 0.001, $\eta_{p}^{2}\;$ = 0.060, indicating that the effect of character style on passion differed substantially between genders.

**Figure 3:**
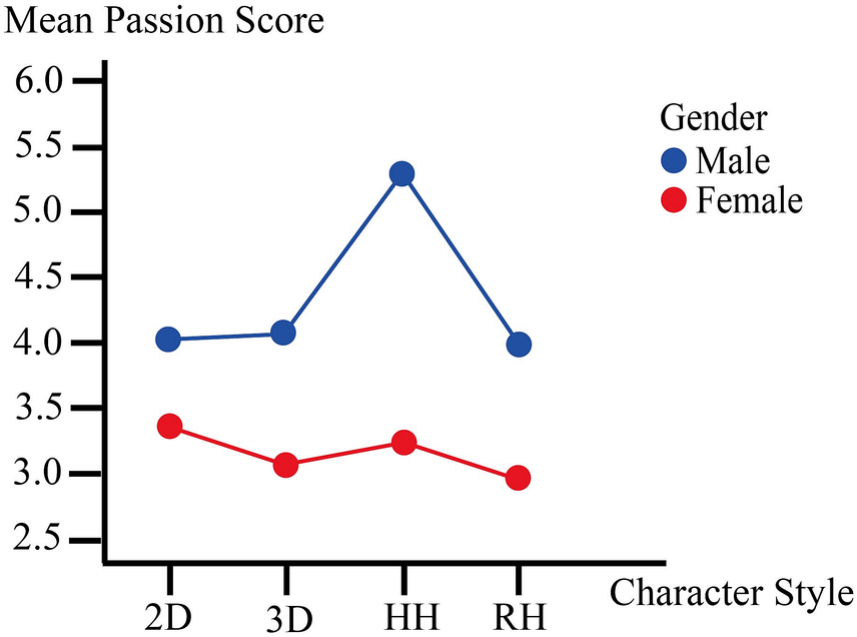
Interaction effect of character style × participant gender on passion. 2D = 2D Anime; 3D = 3D Cartoon; HH = Highly Humanoid; RH = Real Human.

Simple effects analyses showed that character style did not significantly affect passion ratings among female participants, *F* (3, 396) = 1.791, *p* = 0.150; female participants reported comparably low passion scores across all four conditions (range of means: 3.00–3.37). By contrast, a highly significant simple effect was found among male participants, *F* (3, 396) = 11.785, *p* < 0.001. Conditional pairwise comparisons showed that male participants rated Highly Humanoid targets (*M* = 5.35, *SD* = 1.74) substantially higher in passion than 2D Anime (*M* = 4.00, *SD* = 2.07, *p* = 0.002), 3D Cartoon (*M* = 4.04, *SD* = 1.64, *p* < 0.001), and Real Human targets (*M* = 3.91, *SD* = 1.59, *p* < 0.001). No significant differences were found among the remaining three conditions for male participants (Figure 3).

Overall, unlike Trust and Commitment, both Intimacy and Passion showed significant Character Style × Gender interactions, supporting H4. The patterns were complementary: female participants reported elevated intimacy specifically toward 2D Anime targets, while male participants reported elevated passion specifically toward Highly Humanoid targets. Neither interaction followed the simple gradient from low to high realism; instead, each gender showed a pronounced peak at a different point along the character-style continuum. These results support H3 (character style significantly influenced intimacy and passion) and H4 (these effects varied by gender), and are consistent with the second concern outlined in the Introduction—that different character styles may differentially intensify specific dimensions of affective engagement.

## 4 Discussion

### 4.1 Summary of key findings

This study examined how character style and participant gender shape initial romantic evaluations of AI-generated and real human targets across four relational dimensions. The results revealed two distinct patterns that map onto the two-concern framing outlined in the Introduction.

First, on the foundational relational dimensions of Trust and Commitment, the Real Human condition was consistently rated higher than all three AIGC conditions, and no significant Character Style × Gender interaction was observed for either dimension. These findings support H1 and H2 and suggest that, in the present brief-exposure paradigm, AI-generated characters—regardless of their position along the character-style continuum—did not approach the relational standing afforded to a real human target on judgments involving credibility and long-term partnership potential.

Second, on the affective engagement dimensions of Intimacy and Passion, significant Character Style × Gender interactions emerged for both constructs. Female participants reported elevated intimacy specifically toward 2D Anime targets, while their passion ratings remained comparably low across all conditions. Male participants, by contrast, reported similar levels of intimacy across conditions but showed a pronounced elevation in passion toward Highly Humanoid targets. These findings support H3 and H4 and indicate that character style can differentially intensify specific affective responses in ways that vary by gender, though these effects should be interpreted in the context of the present stimulus configurations rather than attributed to visual realism alone (see Limitations).

Taken together, the findings suggest that Trust and Commitment function as relational boundaries that AI-generated characters do not readily cross under brief initial exposure, whereas Intimacy and Passion are more susceptible to character-style and gender-specific variation—a pattern that carries implications for understanding both the limits and the engagement potential of AI companionship technologies. The sections that follow discuss each pattern in turn.

### 4.2 The relational boundary: trust and commitment

The Real Human condition received significantly higher ratings than all three AIGC conditions on Trust, and higher ratings than at least two AIGC conditions on Commitment. Neither dimension showed a significant Character Style × Gender interaction, indicating that this pattern was consistent across male and female participants. These results are consistent with H1 and H2 and suggest that, under brief initial exposure, AI-generated characters did not approach the relational standing afforded to a real human target on foundational relational judgments.

This finding can be understood through the lens of two distinct but converging literatures. In the trust domain, the present items assessed whether participants perceived the target as credible, trustworthy, and capable of sustaining mutual trust. These appraisals draw on both the automation trust tradition, in which trust depends on perceived reliability, predictability, and appropriate reliance (Hoff and Bashir, 2015), and the interpersonal trust tradition, in which trust is grounded in reciprocity, vulnerability, and expectations about another’s intentions under conditions of risk (Rempel et al., 1985; Thielmann and Hilbig, 2015). Recent work on human–AI intimacy suggests that, over extended use, trust in AI companions can develop in a functional or affective form—grounded in the AI’s predictability, emotional stability, and nonjudgmental availability—without requiring the deeper mutual vulnerability characteristic of interpersonal trust (Huang and Lan, 2025). The present findings do not contradict this account but rather reveal what happens when AI-generated targets are evaluated alongside a real human baseline. In this comparative context, real human targets carry interpersonal trust cues—such as biological familiarity, perceived social accountability, and the potential for genuine reciprocity—that AI-generated characters, regardless of their position along the character-style continuum, do not yet convey. The trust gap observed here therefore does not indicate that AI characters are incapable of eliciting any trust, but rather that the form of trust they may support remains distinguishable from, and rated lower than, the trust afforded to a real human target under initial evaluative conditions. Whether extended interactive exposure could allow functional trust in AIGC characters to narrow this gap remains an open question for future research. Notably, the three AIGC conditions did not differ significantly from one another on trust, suggesting that the trust gap observed here reflects a categorical distinction between human and non-human targets rather than a gradient along the character-style continuum.

In the commitment domain, the present items asked whether participants would consider the target as a long-term partner or potential spouse. As discussed in the Measures section, these items were designed as a boundary test rather than as literal expectations, and the consistently low ratings for AIGC conditions can be interpreted as a theoretically informative finding: participants were willing to extend commitment-related evaluations to real human targets but not to AI-generated characters, even highly realistic ones. This pattern aligns with recent reviews noting that AI companions fundamentally lack the reciprocity and shared vulnerability that underpin human commitment (Ho et al., 2025; Muldoon and Parke, 2025). In human romantic relationships, commitment reflects a willingness to accept obligation, durability, and future-oriented responsibility (Stanley and Markman, 1992; Sternberg, 1997). In the absence of perceived reciprocal agency—that is, the sense that the other party can independently choose to remain, sacrifice, or share risk—participants appear to have withheld commitment judgments from AI-generated targets regardless of character style.

Two important qualifications are necessary. First, the trust and commitment ratings obtained here reflect initial dispositional appraisals formed under brief exposure rather than mature relational states. As discussed in the Introduction, thin-slice research demonstrates that such appraisals can be meaningful and internally consistent (Ambady and Rosenthal, 1992), but they necessarily set a ceiling on the relational depth that can be assessed. The relational boundary observed here should therefore be understood as a property of the present brief-exposure paradigm rather than as a fixed limit on human–AI relational potential. Second, the significant main effect of gender on both dimensions (with male participants reporting higher scores overall) indicates that men and women differed in how generously they rated targets in general, but not in which condition they rated highest. In other words, gender influenced the overall level of trust and commitment ratings but did not alter the consistent advantage of the Real Human condition over AIGC conditions.

### 4.3 Gendered affective engagement: intimacy and passion

In contrast to Trust and Commitment, both Intimacy and Passion showed significant Character Style × Gender interactions, indicating that the effects of character style on affective engagement differed between female and male participants. These interactions did not follow a simple linear gradient along the character-style continuum; instead, each gender showed a distinct peak at a different point. Importantly, the observed gender differences may partly reflect responses to the specific stimulus configurations used in this study—including profile cues and voice characteristics—rather than to visual style alone, a point that is addressed more fully in the Limitations section.

Among female participants, the most notable finding was the elevated intimacy reported toward 2D Anime targets, which was significantly higher than toward both 3D Cartoon and Real Human targets. This pattern resonates with research on parasocial relationships—one-sided emotional bonds that audiences form with media figures (Horton and Wohl, 1956)—and their extension into anime and interactive fiction contexts. Qualitative work on female players of Otome games suggests that the bonds formed with stylized 2D characters are not detached from reality but are deeply embedded in players’ emotional lives through idealized interaction and internalized imagination (Gao et al., 2025). Stylized characters may function as emotionally accessible relational targets that offer perceived warmth and closeness without the social evaluation pressures or physical risks that can accompany real-world heterosexual encounters (Skjuve et al., 2022; Li and Zhang, 2024). The elevated intimacy observed here may therefore reflect the cultural familiarity and emotional safety that 2D characters carry for young female users who are regular consumers of anime and interactive fiction content. At the same time, female participants’ passion ratings remained comparably low across all four conditions (range of means: 3.00–3.37), with no significant simple effect of character style. This asymmetry between intimacy and passion suggests that the two dimensions are activated through different pathways. While a brief multimodal introduction was sufficient to elicit closeness-related judgments among female participants, it appears to have been insufficient to activate the kind of romantic or physical attraction that the passion items capture—responses that, for this group, may depend more heavily on sustained narrative engagement, emotional investment, and relational depth than a single 30-s exposure can provide (Potdevin et al., 2021).

The pattern for male participants was markedly different. Passion ratings were substantially higher for the Highly Humanoid condition than for all other conditions, including Real Human—the only instance in the present data in which an AIGC condition surpassed the human baseline on any dimension. One plausible interpretation is that highly humanoid AI-generated characters can be produced with idealized physical features—optimized facial proportions, skin quality, and body presentation—that many viewers find immediately appealing (Tao et al., 2025). Unlike real human targets, who carry natural physical variation and minor imperfections, the Highly Humanoid condition combines a near-human appearance with a degree of aesthetic optimization that is difficult to achieve in reality. Recent evidence supports this account, showing that higher-realism avatars are generally perceived as more attractive, particularly when they approximate but do not fully replicate human appearance (Tao et al., 2025). In addition, the perceived artificiality of the target may have provided a form of psychological safety, allowing participants to experience visual attraction without the social or evaluative risks that accompany real interpersonal encounters (Skjuve et al., 2022). The contrasting passion patterns between genders suggest that different pathways may underlie this dimension. This is consistent with evidence that gender moderates how people form affective responses to media characters, with the nature of the response depending on both the character’s presentation and the evaluative context (Tukachinsky et al., 2020). Related work further suggests that the gendering of AI itself shapes humanness perceptions, with female-presenting AI characters eliciting greater perceived warmth and emotional accessibility (Borau et al., 2021). For male participants, the immediate visual attractiveness of the idealized Highly Humanoid target appears to have been sufficient to elicit a strong passion response within a brief exposure. For female participants, by contrast, passion may depend more on sustained emotional narrative and relational context that a 30-s non-interactive presentation could not provide. This asymmetry likely reflects what the present paradigm was able to activate rather than an inherent difference in the capacity for passion. Finally, male participants showed no significant variation in intimacy across conditions, suggesting that emotional closeness was less responsive to character presentation for this group and may depend on factors not manipulated here, such as interaction depth or conversational reciprocity.

Taken together, the Intimacy and Passion results show that AI-generated characters can elicit meaningful affective responses, but that the nature of these responses is shaped by both character style and participant gender in specific and non-uniform ways. Female participants responded with elevated intimacy to a culturally familiar, stylized character type, while male participants responded with elevated passion to a character type offering idealized near-human appearance. Neither pattern followed a simple realism gradient. These findings are consistent with the second concern outlined in the Introduction—that certain character styles may differentially intensify affective engagement in ways relevant to emotional reliance and technology dependence—while also underscoring the stimulus-specific nature of these effects and the need for caution in generalizing them beyond the present experimental conditions.

### 4.4 Theoretical implications

The present study offers two theoretical contributions to the literature on human–AI romantic interaction. The first is methodological. By combining trust assessment from the HCI and automation literature with Sternberg’s theory, the study provides a cross-disciplinary approach for exploring the relational boundary between AI-generated and real human targets. This dual-framework structure proved informative in the present data: the Trust and Commitment dimensions patterned differently from Intimacy and Passion, both in their sensitivity to character style and in their interaction with participant gender. Had only one family of constructs been measured, this asymmetry would have gone undetected. The approach may be applicable to future studies that seek to map where the relational boundary between AI companions and real humans lies across different evaluative dimensions.

The second contribution is conceptual. The results reveal that the four relational dimensions do not respond to AI-generated characters in a uniform way. Trust and Commitment remained anchored to the Real Human baseline regardless of character style or participant gender, whereas Intimacy and Passion were selectively intensified by specific character configurations in gendered ways. This pattern of dimensional separation suggests that AI companionship does not move relational judgments as a single block; rather, affective engagement dimensions can shift independently of foundational relational dimensions. This distinction between bounded and malleable dimensions may help organize future research on AI companionship, which has often treated relational outcomes as broadly positive or broadly negative without differentiating among them (Ho et al., 2025; Song et al., 2022).

### 4.5 Practical implications

The uneven pattern of findings across dimensions carries different practical implications depending on which dimension is considered. Regarding Trust and Commitment, the present results suggest that users readily distinguish between AI-generated and real human targets on foundational relational judgments, even under conditions designed to present the AI characters in a favorable light. This provides some reassurance that, at least in the context of brief initial encounters, users are not extending the relational evaluations reserved for real partners to AI-generated characters. Designers and policymakers should nonetheless remain attentive to the possibility that extended, interactive AI systems—particularly those with emotional memory and adaptive dialogue—could gradually erode this boundary over time (Ho et al., 2025).

Regarding Intimacy and Passion, the gendered patterns observed here raise a different set of considerations. The finding that specific character styles can selectively intensify affective responses suggests that design choices—such as the degree of stylization, the level of physical idealization, and the emotional tone of character presentation—are not neutral with respect to user engagement. The present data do not support the claim that AI-generated characters are replacing humans across core relational dimensions. They do, however, provide initial evidence that certain character configurations can intensify affective engagement in gendered ways that may be relevant to emotional reliance and technology dependence (Ho et al., 2025; Xie et al., 2023). Rather than recommending that developers exploit these pathways to maximize user retention, the present findings underscore the importance of designing AI companions that are emotionally supportive without becoming manipulative (Muldoon and Parke, 2025). Two design principles follow from these findings. First, the gendered affective patterns suggest that character style choices in AI companion design are not psychologically neutral. The same product may elicit quite different emotional responses from different user groups: stylized characters may foster feelings of closeness among some users, while highly realistic characters may intensify attraction among others. Designers who are aware of these differential effects are better positioned to anticipate—and where necessary, mitigate—the risk that specific character configurations could foster one-sided emotional reliance in vulnerable user populations. Second, the finding that Trust and Commitment remained anchored to the Real Human baseline offers some reassurance that a clear relational boundary currently exists. However, the selective intensification of Intimacy and Passion observed here suggests a possible pathway through which prolonged exposure to affectively engaging AI characters could gradually deepen emotional attachment, even before that boundary is crossed. Whether this pathway leads to sustained dependence, and under what conditions, is a question that longitudinal research will need to address.

### 4.6 Limitations and future research

Several limitations should be considered when interpreting the present findings.

First, the study relied on 30-s video presentations without interactive exchange. This thin-slice paradigm was sufficient to produce reliable initial impressions and meaningful between-condition differences, but it necessarily constrains the relational depth that can be assessed. The trust and commitment ratings obtained here represent initial dispositional appraisals, not mature relational states. Future research should examine whether extended, interactive exposure to AI companions—particularly systems capable of sustained dialogue and emotional memory—would narrow the gap between AIGC and real human targets on these foundational dimensions, or whether the boundary observed here proves durable even under richer interaction conditions.

Second, the study was not designed as a pure manipulation of visual style. As described in the Experimental Design and Stimuli sections, each character condition was a multimodal configuration that included visual appearance, voice, and brief profile cues. Female characters were assigned art-related majors and male characters sports-related majors, and the voice models were graduated to match each style’s level of realism. While these design choices were made to ensure within-style coherence and ecological plausibility, they introduce the possibility that the observed effects—particularly the gendered patterns in Intimacy and Passion—were partly driven by profile content or voice quality rather than by visual style alone. Future studies could address this by systematically crossing visual style with profile and voice conditions, though this would require a substantially larger stimulus set.

Third, the Real Human condition differed from the AIGC conditions not only in visual appearance but also in voice modality (natural human recording vs. TTS synthesis). This difference was preserved deliberately to maintain the ecological authenticity of the human baseline, but it means that the trust and commitment advantage observed for Real Human targets cannot be attributed solely to visual presentation. Future designs might include a condition in which a real human target is paired with synthetic voice, or vice versa, to disentangle the contributions of visual and auditory modality.

Fourth, the sample consisted of 134 Chinese university students aged 17–24, all evaluated within a heterosexual romantic framework. The findings may not generalize to older age groups, non-heterosexual orientations, or cultural contexts with different norms around AI companionship and romantic relationships. Given that the user base for AI companion technologies is rapidly diversifying in both age and cultural background, extending this research to more heterogeneous samples is an important priority.

Fifth, the cross-sectional design captured only immediate responses. It remains unknown whether the affective engagement patterns observed here—such as the elevated passion toward Highly Humanoid targets among male participants—would persist, intensify, or diminish with repeated exposure. Longitudinal designs that track users’ relational evaluations of AI characters over weeks or months would be valuable for assessing whether selective affective engagement translates into sustained emotional dependence. Future work might also explore whether design interventions—such as AI characters that occasionally express differing perspectives or set interactional boundaries—could mitigate the risk of one-sided emotional reliance.

## 5 Conclusion

This study examined how character style and participant gender shape initial romantic evaluations of AI-generated and real human targets across four relational dimensions. The results reveal a consistent pattern: Trust and Commitment remained higher for real human targets across all conditions and both genders, whereas Intimacy and Passion were selectively influenced by character style in gendered ways—with female participants reporting elevated intimacy toward stylized 2D Anime characters and male participants reporting elevated passion toward Highly Humanoid characters.

These findings suggest that, under brief initial exposure, AI-generated characters do not approach real humans on the foundational relational dimensions of trust and commitment, but they can elicit meaningful affective responses on the dimensions of intimacy and passion. The relational boundary between AI and human targets thus appears to be dimension-specific rather than absolute: firmly held on some evaluative dimensions and more permeable on others. Understanding where this boundary lies, how it shifts across character configurations and user groups, and whether it erodes with extended interaction are questions that will become increasingly important as AI companion technologies continue to develop.

## Data Availability

The raw data supporting the conclusions of this article will be made available by the authors, without undue reservation.
